# Provision of Psychodynamic Psychotherapy in Austria during the COVID-19 Pandemic: A Cross-Sectional Study

**DOI:** 10.3390/ijerph18179046

**Published:** 2021-08-27

**Authors:** Andrea Jesser, Johanna Muckenhuber, Bernd Lunglmayr, Rachel Dale, Elke Humer

**Affiliations:** 1 Department for Psychotherapy and Biopsychosocial Health, Danube University Krems, 3500 Krems, Austria; rachel.dale@donau-uni.ac.at (R.D.); elke.humer@donau-uni.ac.at (E.H.); 2 Institut für Soziale Arbeit, FH Joanneum University of Applied Science, 8020 Graz, Austria; johanna.muckenhuber@fh-joanneum.at; 3 Research Workgroup, Austrian Society for Applied Depth Psychology and Psychotherapy (ÖGATAP), 1150 Vienna, Austria; kontakt@lunglmayr.at; 4 Doctorate in Psychotherapy by Professional Studies Programme, Metanoia Institute, Ealing, London W5 2QB, UK

**Keywords:** psychotherapy, psychodynamic, telephone, videoconferencing, attitudes, COVID-19

## Abstract

The COVID-19 pandemic has brought massive changes in the provision of psychotherapy. To avoid or reduce the risk of infection, many therapists switched from face-to-face sessions in personal contact to remote psychotherapy, i.e., psychotherapy delivered by telephone or videoconferencing. This study examined the attitudes toward and practice of remote psychotherapy among Austrian therapists with a psychodynamic orientation at the onset of the pandemic as well as changes in the therapeutic process that were experienced by the therapists due to switching to a remote setting. A total of 161 therapists with psychodynamic orientation took part in an online survey. The results show that attitudes toward remote psychotherapy changed positively in psychodynamically orientated therapists and most are willing to switch to remote settings, if necessary. However, many therapists reported negative effects of remote psychotherapy and prefer seeing their patients in-person. The strongest changes were experienced with regard to transference/countertransference, the therapeutic process and the intensity of session. The analysis further revealed an overall decrease in the number of patients treated, indicating an undersupply of psychotherapy, at least during the first wave of COVID-19 infection in Austria. In summary, the experience during the first COVID-19 lockdown has led to an increase in remote psychotherapy and more openness toward these treatment modalities among psychodynamically oriented therapists. However, in-person therapy will remain the first choice for most therapists.

## 1. Introduction

The COVID-19 viral pandemic and its associated stressors, such as curfews, social distancing measures, job loss and self-quarantine, significantly impact the human psyche [1]. In Austria, the first COVID-19 cases were reported at the end of February 2020. Like many other countries, the Austrian government introduced obligatory COVID-19 lockdown measures in an attempt to contain the spread of the virus. From 16 March 2020 until 30 April 2020, a nationwide curfew entailed restrictions in movement and activities with few exceptions, such as meeting necessary basic needs of daily life, fulfilling work responsibilities and outdoor activities alone or with people from the same household. Like other healthcare treatments, psychotherapy was included in the few exceptions from the full lockdown restrictions. However, general precautions, such as keeping a safe distance between people, had to be maintained. Although possible, in-person psychotherapy sessions were strongly reduced in Austrian psychotherapists during the early weeks of the lockdown, to mitigate the risk of infection for both psychotherapists and patients [2]. Moreover, most insurances started reimbursing the costs for remote psychotherapy with the first COVID-19 lockdown, which likely further facilitated the provision of remote psychotherapy [3].

A study conducted at the beginning of the COVID-19 pandemic in Austria (April 2020) on a representative sample of the Austrian general population revealed a strong increase in mental health problems compared to pre-pandemic studies. High prevalences of mental health disorders were observed, such as 21% for depression, 19% for anxiety and 16% for insomnia [4]. About 10 months after the COVID-19 outbreak in Austria, during the second wave of COVID-19 infections (December 2020-January 2021), a further increase in mental health disturbances was observed (26% depression, 23% anxiety, 18% insomnia) [5], indicating an increased need for mental health care during the COVID-19 pandemic.

To meet the increased need for psychotherapeutic support, while adhering to the efforts to contain the spreading of the virus, i.e., via reducing physical contacts, the use of alternative psychotherapy formats, such as psychotherapy via videoconferencing or telephone, seems to be a valuable option [6]. Several studies highlight that psychotherapists are in general more critical about remote psychotherapy settings than their patients [7]. A large study among more than 1500 Austrian psychotherapists conducted during the first weeks of the COVID-19 lockdown in Austria reported that, regardless of the therapeutic orientation, psychotherapists experienced remote psychotherapy better than expected, but not fully comparable to face to face psychotherapy in personal contact [8]. In general, previous studies investigating remote psychotherapy settings revealed that remote psychotherapy can be as effective as in-person psychotherapy, and even therapeutic alliance can be as good as in psychotherapies conducted in personal contact [9,10,11,12,13]. Most of these studies involved cognitive behavioral therapy, while less research has investigated psychodynamically-oriented psychotherapies. The concurrent rise of remote psychotherapy as an alternative treatment modality requires further analysis of its impact in a psychodynamic psychotherapy setting. 

In Austria about one quarter of licensed psychotherapists are practicing psychodynamic psychotherapy [14]. This corresponds to 2595 active therapists. The psychodynamic orientation comprises psychoanalytic methods and methods oriented toward depth psychology. The current study was supported by an Austrian Society offering training in different methods oriented toward depth psychology (Guided affective Imagery, Hypnosis, and Autogenous Relaxation). A cross-sectional online survey was conducted in May 2020 among members of the Society to investigate remote psychotherapy settings in psychodynamic psychotherapy during the COVID-19 pandemic in Austria. The study aimed to investigate the format psychotherapy was delivered by psychotherapists with psychodynamic orientation at the beginning of the COVID-19 pandemic and how they planned to deliver psychotherapy after the lockdown and in the future. We further aimed to investigate the attitudes of psychodynamically-orientated psychotherapists towards remote psychotherapy and possible changes due to the COVID-19 pandemic. We were also interested in their subjective experiences providing psychotherapy by telephone or videoconferencing and how these experiences are reflected in the existing general and psychodynamically influenced discussion around remotely delivered psychotherapy. Moreover, the study aimed to explore which patients were most willing to switch to remote sessions from the therapists’ perspective.

## 2. Materials and Methods

### 2.1. Study Design

An online survey was designed in LimeSurvey Professional [15], comprising 69 items in total. Austria has a long tradition of psychotherapy dating back to the 1920s and a broad spectrum of established psychotherapeutic methods. Psychotherapists can choose out of 23 accredited psychotherapeutic methods: (1) Analytical Psychotherapy, (2) Group Psychoanalysis, (3) Individual Psychology, (4) Psychoanalysis/Psychoanalytical Psychotherapy, (5) Psychoanalytically oriented Psychotherapy, (6) Autogenous Relaxation, (7) Daseinsanalysis, (8) Dynamic Group Psychotherapy, (9) Hypnosis, (10) Guided Affective Imagery, (11) Concentrative Movement Therapy, (12) Transactional Analysis, (13) Existential Analysis, (14) Existential Analysis and Logotherapy, (15) Gestalttheoretical Psychotherapy, (16) Integrative Gestalttherapy, (17) Integrative Therapy, (18) Client-centred Psychotherapy, (19) Person-centred Psychotherapy, (20) Psychodrama, (21) Neuro-linguistic Psychotherapy, (22) Systemic Family Therapy and (23) Behavioural Therapy [16]. These 23 methods can be categorized into four theoretical orientations: humanistic (about 33% of licensed psychotherapists), psychodynamic (about 28% of licensed psychotherapists), systemic (about 26% of licensed psychotherapists) and behavioral (about 13% of licensed psychotherapists) [14]. 

This cross-sectional study was supported by the Austrian Society for applied Depth Psychology and Psychotherapy (ÖGATAP), which offers basic and advanced training for three methods belonging to the psychodynamic orientation: Guided Affective Imagery, Hypnosis, and Autogenous Relaxation. Together, these methods account for one third of therapists (*n* = 814) with psychodynamic orientation. The ÖGATAP sent a link to the online survey to all registered members (*n* = 687 with a respondence rate of 23.4%). With *n* = 161 respondents, 19.8% of officially registered therapists of the mentioned psychodynamic methods participated in the survey.

The survey was open from 5 May 2020 to 25 May 2020. Participation was voluntary, without incentives. Participants had to agree to the data protection declaration to start the survey (electronic informed consent). The principles outlined in the Declaration of Helsinki were followed and the ethics committee of the ÖGATAP (Vienna, Austria) approved the study.

### 2.2. Measures

Psychotherapists were asked about the following topics:

- Psychodynamic orientation in which patients are treated: Guided Affective Imagery, Hypnosis, Autogenous Relaxation, others
- Format they are currently providing psychotherapy (videoconferencing, telephone, in-person with and without additional safety measures)
- Provision of remote psychotherapy (videoconferencing, telephone) before COVID-19 (no, often, from time to time, rarely)
- Attitudes toward remote psychotherapy (videoconferencing, telephone) before COVID-19 (retrospectively) as well as in the current situation (5-point scale from 1 “very good” to 5 “very critically”)
- Number of adult patients, adolescents and children treated before the COVID-19 lockdown
- Number of adult patients, adolescents and children treated since the COVID-19 lockdown (16 March 2020) via videoconferencing, via telephone, in personal contact with and without additional safety measures
- Number of therapies per patient group (adult patients, adolescents, children) suspended and terminated since the COVID-19 lockdown
- Planned psychotherapy format directly after the lockdown
- Planned psychotherapy format until the end of the COVID-19 pandemic
- Planned psychotherapy format after the COVID-19 pandemic
- Perception of any limitations/difficulties or benefits of remote psychotherapy (a number of free-text questions were also included; however, results of this analysis will be published separately)
- Rating of potential experienced changes through the remote setting (videoconferencing, telephone) concerning seven aspects (therapeutic alliance, therapeutic process, content of sessions, patients’ and therapists’ contribution to the therapeutic dialogue, intensity of sessions, structure of sessions, transference-countertransference) on a 5-point scale from 1 “very strong change” to 5 “no change”
- Estimated willingness of different groups of patients to switch to remote psychotherapy (telephone or videoconferencing) on a 5-point scale from 1 “very high willingness” to 5 “very low willingness” for the following groups: patients with psychiatric disorders; clients without psychiatric disorders, e.g., clients undergoing psychotherapy for personality development; specific groups of psychiatric diagnosis (delusional disorders, affective disorders, somatoform disorders, personality disorders); and different levels of personality structure (high, medium, low)—these categories were derived from a psychodynamic model used to organize disorders along a structural continuum of severity, with high referring to a neurotic level (i.e., the healthiest level of personality organization, describing people with intact reality testing, a consistent sense of self and others and mature defense mechanisms), low referring to a borderline level (i.e., a low level of personality organization, describing people with difficulties with reality testing, an inconsistent sense of self and others and primitive defense functioning), and medium referring to the level at the transition between the neurotic and borderline level [17].

### 2.3. Statistics 

Statistical analyses were performed with SPSS Version 26 (IBM Corporation, Armonk, NY, USA). Frequencies and percentages were calculated to describe the sample. 

Potential changes in the total caseload, i.e., the number of patients treated since the COVID-19 lockdown vs. before the COVID-19 lockdown, were investigated by a *t*-test for dependent samples. To investigate whether this change in the number of treated patients interacted with the patient group (adults, adolescents, children), a mixed ANOVA (RM-ANOVA) was computed.

Potential differences in the number of adult patients, adolescents and children treated per treatment format (in-person, videoconferencing, telephone) since the COVID-19 lockdown were analyzed by RM-ANOVA. 

Further RM-ANOVAs were calculated to investigate whether changes in the number of suspended or terminated treatments since the COVID-19 lockdown interacted with the patient group (adults, adolescents, children).

To investigate whether the attitudes towards remote psychotherapy changed as compared to before the pandemic, a *t*-test for dependent samples was computed. *T*-tests for independent samples were computed to investigate whether it made a difference for therapists’ attitudes toward remote psychotherapy if someone had worked remotely before the pandemic.

RM-ANOVAs were performed to investigate the perceived changes of seven different aspects of psychotherapy (therapeutic alliance, therapeutic process, content of sessions, patients’ and therapists’ contribution to the therapeutic dialogue, intensity of sessions, structure od sessions, transference-countertransference) due to the switch to remote psychotherapy.

To investigate psychotherapists’ experience of whether patients with psychiatric disorders were more willing to change to remote settings as compared to patients without a psychiatric disorder, a *t*-test for dependent samples was computed.

Moreover, an RM-ANOVA was performed to investigate differences in the experienced willingness to change to remote settings concerning three levels of personality structure (low, medium, high). A further RM-ANOVA was computed to investigate differences in the experienced willingness to change to remote settings concerning four different diagnosis groups (delusional disorders, affective disorders, psychosomatic disorders, personality disorder).

All main effects (ME) and interaction effects (IE) of the RM-ANOVAs were examined. The Greenhouse-Geisser corrected values are presented. Bonferroni corrections were applied for the pairwise post-hoc tests.

All statistical analyses were two-tailed, with a *p*-value < 0.05 indicating statistical significance.

## 3. Results

### 3.1. Study Sample

In total, *n* = 161 psychotherapists completed the online survey. 81.4% were female (compared to 77.9% female psychotherapists practicing Guided Affective Imagery, Hypnosis and Autogenous Relaxation, according to the Austrian list of psychotherapists). Sociodemographic characteristics are summarized in Table 1.

### 3.2. Changes in the Provision of Psychotherapy Due to the COVID-19 Lockdown

A total of 128 (79.5%) therapists provided psychotherapy via videoconferencing since the start of the COVID-19 lockdown. The same number of therapists reported providing psychotherapy via telephone. While 104 therapists (64.6%) provided in-person psychotherapy with safety measures, only three psychotherapists (1.9%) stated that they provide in-person psychotherapy the same way as they did before the pandemic (without additional safety measures). Due to the low number of psychotherapists providing in-person psychotherapy without additional safety measures, they were categorized together with those applying safety measures into the category “in-person psychotherapy” for further analyses.

The total number of patients (adult + adolescents + children) treated before the pandemic per psychotherapist, i.e., the total caseload, (M = 15.20, SD = 8.62) decreased to 11.87 (SD = 8.80) during the pandemic, t(1, 160) = 6.974; *p* < 0.001, referring to a decrease of 21.9%. 

Results separated by the patient group are summarized in Table 2. An overall decrease in the number of patients treated (ME “change” = *p* < 0.001), as well as an overall difference in the number of patients treated concerning patient group (ME “patient group” = *p* < 0.001) was observed. Pairwise post-hoc tests revealed higher numbers of adult patients treated compared to adolescents (*p* < 0.001) and children (*p* < 0.001), as well as a higher number of adolescents treated as compared to children (*p* = 0.002). An interaction between the change in the number of patients treated and patient group was observed (IE “change × patient group”; *p* < 0.001), revealing a decrease in adult patients treated since the lockdown compared to before the lockdown by on average 21.1% (*p* < 0.001; Table 2). No significant difference was observed for the number of adolescents (*p* = 0.133), whereas the number of treated children decreased by 44.4% (*p* = 0.003). 

A more detailed look at the format in which psychotherapy has been provided since the COVID-19 lockdown, differentiated per patient group, is summarized in Table 3. Results of the RM-ANOVAs revealed an overall difference in the patients treated per group (ME “patient group” = *p* < 0.001), as well as an overall difference in the number of patients treated per treatment format (ME “format” = *p* = 0.010). Post-hoc tests revealed higher numbers of patients treated by video conferencing as compared to in-person (*p* = 0.017), but no difference between video conferencing and telephone (*p* = 0.131), nor between telephone and in-person (*p* = 0.778). An interaction between the format and patient group was observed (IE “format x patient group”; *p* = 0.015), revealing significant differences in the treatment format in adults, but not in the other patient groups. More specifically, a 52% higher number of adults were treated via videoconferencing as compared to in-person (*p* = 0.021), whereas post-hoc tests revealed no difference between videoconferencing and telephone (*p* = 0.170), nor between telephone and in-person psychotherapy (*p* = 0.697).

The proportion of suspended and terminated psychotherapies in relation to the number of treated patients before the COVID-19 lockdown per patient group are summarized in Table 4. RM-ANOVAs, taking only those therapists into account who treated patients of all groups before the pandemic (*n* = 25), revealed no significant difference in the number of suspended (*p* = 0.201) as well as terminated (*p* = 0.361) therapies per patient group. However, on a purely descriptive level differences in percentages can be seen.

### 3.3. Provision of Psychotherapy after the COVID-19 Lockdown

Therapists were asked whether they intend to change to in-person psychotherapy after the end of the COVID-19 lockdown (end of April 2020). In total 13.0% of the psychotherapists stated that they are already treating solely in-person (Table 5). 36.6% stated that they will switch all remote psychotherapies to in-person psychotherapy and another 36.6% psychotherapists stated that they will partially switch to in-person psychotherapy. The minority of psychotherapists (13.7%) stated that they do not want to switch to in-person psychotherapy immediately after the end of the COVID-19 lockdown.

Therapists who stated that they will provide in-person psychotherapy after the lockdown (*n* = 139), were further asked about the way they will conduct in-person sessions concerning safety measures. The majority of therapists (98.6%) stated that they will keep a safe distance of 1 m and regularly disinfect all surfaces in the practice (91.4%; Table 6). In total 62.6% psychotherapists stated that they will apply other safety measures such as keeping the window open or wearing a face shield. Less than one fifth of the therapists stated that they will wear a mask or want their patients to wear a mask. Not a single therapist stated to apply no additional safety measures.

Overall, the majority of therapists stated that they will offer remote psychotherapy until the end of the pandemic (Table 7). More specifically, 5.0% stated that they feel more comfortable with remote psychotherapy, 53.4% stated that they will do so if the patient feels more comfortable with it, 35.4% stated that they will do so only if there is no other way and 6.2% stated that they do not want to offer remote psychotherapy.

Finally, 18.0% of the therapists stated that they do not want to use remote psychotherapy after the end of the COVID-19 pandemic, and 26.1% stated they want to use remote psychotherapy after the pandemic. Overall, the majority (55.9%) stated that they will do so if necessary.

### 3.4. Attitudes toward Remote Psychotherapy

Attitudes towards remote psychotherapy improved during the COVID-19 situation (M = 2.33, SD = 1.166) as compared to the time before the pandemic (M = 3.68, SD = 1.191) t(1, 160) = 12.832; *p* < 0.001. 

In total 40 therapists (24.8%) reported that they already applied remote psychotherapy (via videoconferencing or telephone) before the pandemic. Among these therapists, three applied remote psychotherapy often, seven from time to time, and the remainder (30) rarely before COVID-19. Attitudes towards remote psychotherapy before the pandemic were higher in therapists with previous experience in remote psychotherapy (M = 3.15, SD = 1.099) as compared to those without (M = 3.86, SD = 1.17; *p* = 0.001), whereas no difference was reported during the pandemic (*p* = 0.840). The change in the attitude (attitude during the pandemic–attitude before the pandemic) differed (*p* = 0.001), with a decrease of −0.850 (SD = 0.949), in those with experience and of −1.521 (SD = 1.409) in those without experience, showing that those without experience showed an even more positive change than those with previous experience. 

In total, 136 (84.5%) psychotherapists stated that they experience limitations/difficulties with remote psychotherapy. The number of psychotherapists who stated that they experience benefits with remote psychotherapy was lower (*n* = 110, 68.3%).

**Figure 1:**
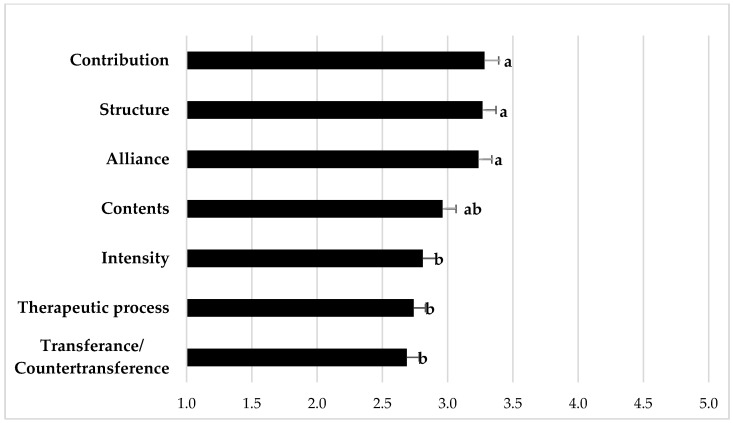
Therapists’ rating of experienced changes through the remote setting (videoconferencing, telephone) concerning the different aspects. Note: Psychotherapists rated their experienced change on a 5-point scale from 1 “very strong change” to 5 “no change”. Data are shown as least square means (LSM) ± standard error of the means. a,b Different superscripts indicate differences among LSM of the topics at *p* < 0.05 after Bonferroni-correction.

Psychotherapists’ ratings of experienced changes through the remote setting per aspect are depicted in Figure 1. Although for all aspects a change was reported, these changes were experienced differently concerning the different aspects of psychotherapy (F(5.279; 0.872) = 11.675; *p* < 0.001). The strongest changes were reported for transference/countertransference, the therapeutic process and intensity, differing from the therapeutic alliance, structure and the patients’ and therapists’ contribution to the therapeutic dialogue (*p* < 0.05 for all pairwise post-hoc tests).

### 3.5. Willingness of Patients to Switch to Remote Psychotherapy

Therapists reported no difference in the experienced willingness to conduct psychotherapy remotely between patients with a definite diagnosis of mental disorders and those without a diagnosis of a mental disorder (t(1,87) = −0.258; *p* = 0.796).

Therapists stated that patients with a low level of personality structure are less willing to participate in remote psychotherapy (M = 3.11, SD = 1.281) as compared to those with a medium (M = 2.40, SD = 0.965) or a high level of personality structure (M = 2.39, SD = 1.245); F(1.420; 1.361) = 15.811; *p* < 0.001). 

Therapists experienced differences in the willingness to move to remote psychotherapy concerning the diagnosis, with the lowest willingness reported for delusional disorders (*p* = 0.031; Table 8). 

## 4. Discussion

This research aimed to explore changes in the provision of psychodynamically oriented psychotherapy during the COVID-19 pandemic in Austria and to explore therapists’ attitudes toward remote psychotherapy.

One major finding was that the COVID-19 lockdown caused an overall decrease in the number of patients treated by 22% on average, with the strongest relative decline in children, whereas no significant decline was observed for adolescents. During the COVID-19 pandemic most adult patients were treated via videoconferencing and the lowest number in person. For adolescents and children, no difference in treatment modality was observed, which might also be due to the lower number of therapists treating adolescents (*n* = 64) and children (*n* = 24) during the time of the survey. Similar results have been observed in other countries, such as the Czech Republic or Slovakia during spring 2020 [18]. However, an earlier study conducted a few weeks earlier in Austria (during the first weeks of the COVID-19 lockdown) revealed that most patients were treated via telephone, followed by videoconferencing. Therefore, it might be that with the prolongation of the COVID-19 pandemic, more patients switched to more advanced treatment formats, such as videoconferencing as compared to telephonic communication. However, the decrease of in-person psychotherapy was not compensated for by the increase of remote psychotherapy and among adult patients, almost 27% of the therapies were suspended, indicating an undersupply of psychotherapy, at least during the first wave of COVID-19 infection in Austria.

Results revealed that the majority of therapists are willing to switch to other therapeutic formats if necessary, especially, when their patients feel more comfortable with remote settings. However, most therapists (86.2%) intended to switch back to in-person psychotherapy after the end of the lockdown, at least partially, indicating that psychotherapy in person is considered the first choice for most therapists with psychodynamic orientation. This is reflected by previous research on remote treatment in psychodynamic therapy. Although therapists consider advantages of therapy via telephone or videoconferencing, many tend to view it as second best to psychotherapy in person [19,20,21]. On the grounds of the theoretical concepts and approaches that constitute the basis of psychodynamic practice, therapists have expressed serious doubt and skepticism regarding the usefulness and effectiveness of remote psychotherapy. In particular, it has been argued that transference manifestations might develop differently in the remote setting [22,23]. Furthermore, there are concerns that communicating via telephone or videoconferencing might affect the therapeutic relationship, as the perception and interpretation of non-verbal communication is severely limited in remote psychotherapy [24,25,26]. Given these findings, it is not surprising that in our study, most respondents (85%) indicated experiencing negative aspects about remote settings. A more specific analysis of different aspects of the psychotherapeutic encounter revealed that transference/countertransference processes, the intensity and the therapeutic process change the most. This is supported by previous studies, revealing that remote sessions were experienced to be more superficial [27]. Specific therapeutic interventions were also rated to be more typical in in-person settings as compared to remote settings [28]. 

Overall, all psychotherapists reported more positive attitudes toward remote psychotherapy during the COVID-19 pandemic as compared to the period before. In particular, those therapists who used remote therapy settings for the first time reported a stronger improvement of their attitudes compared to those with previous experience. This is in line with existing research, indicating a more positive attitude toward remote psychotherapy upon using remote psychotherapy [6,29,30]. Together with these changes in attitude in favor of remote psychotherapy, the majority of therapists (82%) stated that they want to use remote psychotherapy after the end of the COVID-19 pandemic. However, among those who are intending to provide psychotherapy remotely after the pandemic, the majority specified that they are willing to use remote psychotherapy if there is no other way.

This study has several limitations. First, the cross-sectional design might have caused a recall bias regarding the retrospective rating of attitudes toward remote psychotherapy and regarding the number of patients treated before the COVID-19 lockdown. Second, the study was conducted online, which might have caused a respondent bias towards higher participation of therapists with a higher preference for psychotherapy via videoconferencing. Third, all ratings took only the therapist’s perspective into account, while no survey among patients was conducted. Fourth, results might not be generalizable to other countries with a long tradition in remote psychotherapy or different health care systems.

Future studies on psychodynamic psychotherapists’ attitudes toward remote treatment in countries with more developed e-health solutions in psychotherapy are needed. In addition, further studies should also investigate patients’ perspectives. Since the study was conducted at the beginning of the COVID-19 pandemic, it would be interesting to monitor possible changes in the provision of remote psychotherapy as well as in attitudes toward remote treatments with the prolongation of the pandemic.

## 5. Conclusions

In conclusion, the COVID-19 lockdown went along with a lower number of patients treated in total, a change in the format psychotherapy was provided, and with an increase in psychotherapy from distance, i.e., via videoconferencing or telephone. Although attitudes toward remote psychotherapy changed positively in psychodynamically orientated therapists, most of them experienced limitations of remote psychotherapy and stated to prefer in-person psychotherapy in the aftermath of the pandemic. In practice, this means that further initiatives are needed to improve acceptance of remote psychotherapy settings among psychodynamic psychotherapists, not only to ensure timely and safe psychotherapeutic support during the pandemic, but also in the long term to enable access to psychotherapeutic support for individuals who are unable to access face-to-face psychotherapy for logistical or other reasons [31]. 

## Figures and Tables

**Table 1:** Sociodemographic characteristics of the sample.

Characteristics	*n*	%
Gender		
Female	131	81.4
Male	28	17.4
Others	2	1.2
Age		
≤40	23	14.3
41–50	62	38.5
51–60	46	28.6
>60	30	18.6
Years in profession		
≤5	45	28.0
5.1–10	40	24.8
10.1–20	40	24.8
>20	36	22.4
Psychodynamic Orientation ^1^		
Guided affective imagery	132	72.9
Autogenous relaxation	8	4.4
Hypnosis	32	17.7
Others	9	5.0

^1^ Multiple responses were possible.

**Table 2:** Numbers of patients treated before vs. during the COVID-19 lockdown per patient group.

Patient Group	Before COVID-19 Lockdown, M (SD)	During COVID-19 Lockdown, M (SD)	Statistics
Adults	13.34	10.53	ME “change” F (1; 6.137) = 48.639; *p* < 0.001
	(7.66)	(7.88)	
Adolescents	1.24	0.98	ME “patient group” F (1.138; 66.376) = 353.188; *p* < 0.001
	(2.66)	(2.24)	
Children	0.63	0.35	IE “change × patient group”
	(2.13)	(1.65)	F (1.251; 10.392) = 26.522; *p* < 0.001

Note: M = mean; SD = standard deviation; change = since COVID-19 lockdown vs. before COVID-19 lockdown; ME = Main effect; IE = Interaction effect.

**Table 3:** Numbers of patients by treatment format during the COVID-19 pandemic.

Patient Group	M	SD	Statistics
Adults			
Videoconferencing	4.34	4.54	ME “format” F (2; 8.706) = 4.906; *p* = 0.010
Telephone	3.35	4.07	
In-person	2.85	4.77	
			ME “patient group” F (2; 12.732) = 243.736; *p* < 0.001
Adolescents			
Videoconferencing	0.460	1.35	
Telephone	0.286	0.88	IE “format × patient group”
In-person	0.236	0.75	F (2.065; 12.621) = 4.179; *p* = 0.015
Children			
Videoconferencing	0.106	0.46	
Telephone	0.118	0.82	
In-person	0.130	1.22	

Note: M = mean; SD = standard deviation; ME = Main effect; IE = Interaction effect.

**Table 4:** The proportion of treatments suspended and terminated since the COVID-19 lockdown by patient group.

Patient Group	Percentage (SD)	Statistics
Adults		Suspended:
Suspended	26.72 (25.57)	F(1.397; 0.431) = 1.710;
Terminated	1.11 (3.14)	*p* = 0.201
Adolescents		
Suspended	47.02 (82.30)	Terminated:
Terminated	1.33 (0.07)	F(1.292; 0.002) = 0.947;
Children		*p* = 0.362
Suspended	54.44 (42.09)	
Terminated	0.00 (0.00)	

**Table 5:** Psychotherapists’ intention to change to in-person psychotherapy after the end of the COVID-19 lockdown.

	*n*	%
Already switched all remote psychotherapies back to in-person format	21	13.0
Yes, will switch all remote psychotherapies to in-person	59	36.6
Yes, partially	59	36.6
No, not yet	22	13.7

**Table 6:** Psychotherapists’ intended way to provide in-person psychotherapy after the end of the COVID-19 lockdown ^1^.

	*n*	%
I will wear a mask	26	18.7
Patients have to wear a mask	22	15.8
I will keep a safe distance of 1 m	137	98.6
I will disinfect all surfaces in the practice regularly	127	91.4
Other measures (e.g., face shield, keeping the window open)	87	62.6
I will apply no additional safety measures	0	0

^1^ Multiple responses were possible.

**Table 7:** Psychotherapists’ intention to use remote psychotherapy until the end of the COVID-19 pandemic.

	*n*	%
Yes, I feel more comfortable with it	8	5.0
Yes, if the patient feels more comfortable with it	86	53.4
Yes, if there is no other way	57	35.4
No	10	6.2

**Table 8:** Willingness to move to remote psychotherapy by diagnosis.

Diagnosis	M	SD	Statistics
Delusional disorder	3.17	1.581	F(2.143; 1.058) = 3.723;
Affective disorder	2.39	1.243	*p* = 0.031
Somatoform disorder	2.28	1.274	
Personality disorder	2.61	1.290	

Note: Psychotherapists were asked to rate the experienced willingness of patients to switch to remote psychotherapy (telephone or videoconferencing) on a 5-point scale from 1 “very high willingness” to 5 “very low willingness”. M = mean; SD = standard deviation.

## Data Availability

The raw data supporting the conclusion of this article will be made available by the authors upon reasonable request.

## References

[1] Swartz H.A. (2020). The role of psychotherapy during the COVID-19 pandemic. Am. J. Psychother..

[2] Probst T., Stippl P., Pieh C. (2020). Changes in provision of psychotherapy in the early weeks of the COVID-19 lockdown in Austria. Int. J. Environ. Res. Public Health.

[3] Humer E., Thomas P. (2020). Provision of Remote Psychotherapy during the COVID-19 Pandemic. Digit. Psychol..

[4] Pieh C., Budimir S., Probst T. (2020). The effect of age, gender, income, work, and physical activity on mental health during coronavirus disease (COVID-19) lockdown in Austria. J. Psychosom. Res..

[5] Dale R., Budimir S., Probst T., Stippl P., Pieh C. (2021). Mental health during a COVID-19 lockdown over the Christmas period in Austria. SSRN Electron. J..

[6] Humer E., Stippl P., Pieh C., Pryss R., Probst T. (2020). Psychodynamic, humanistic, systemic, and behavioral psychotherapists’ experiences with remote psychotherapy during COVID-19 in Austria: A cross-sectional online survey (Preprint). J. Med. Internet Res..

[7] Connolly S.L., Miller C.J., Lindsay J.A., Bauer M.S. (2020). A systematic review of providers’ attitudes toward telemental health via videoconferencing. Clin. Psychol. Sci. Pract..

[8] Humer E., Sitppl P., Pieh C., Pryss R., Probst T. (2020). Experiences of Psychotherapists With Remote Psychotherapy During the COVID-19 Pandemic: Cross-sectional Web-Based Survey Study. J. Med. Internet Res..

[9] Backhaus A., Agha Z., Maglione M.L., Repp A., Ross B., Zuest D., Rice-Thorp N.M., Lohr J., Thorp S.R. (2012). Videoconferencing psychotherapy: A systematic review. Psychol. Serv..

[10] Bashshur R.L., Shannon G.W., Bashshur N., Yellowlees P.M. (2016). The Empirical Evidence for Telemedicine Interventions in Mental Disorders. Telemed. e-Health.

[11] Jenkins-Guarnieri M.A., Pruitt L.D., Luxton D.D., Johnson K. (2015). Patient perceptions of telemental health: Systematic review of direct comparisons to in-person psychotherapeutic treatments. Telemed. e-Health.

[12] Langarizadeh M., Tabatabaei M.S., Tavakol K., Naghipour M., Rostami A., Moghbeli F. (2017). Telemental health care, an effective alternative to conventional mental care: A systematic review. Acta Inf. Med..

[13] Lopez A., Schwenk S., Schneck C.D., Griffin R.J., Mishkind M.C. (2019). Technology-Based Mental Health Treatment and the Impact on the Therapeutic Alliance. Curr. Psychiatry Rep..

[14] Austrian Federal Ministry of Social Affairs, Health, Care, and Consumer Protection PsychotherapeutInnenliste. http://psychotherapie.ehealth.gv.at/.

[15] Engard N.C. (2009). LimeSurvey http://limesurvey.org. Public Serv. Q..

[16] Heidegger K.E. The Situation of Psychotherapy in Austria. https://www.europsyche.org/app/uploads/2019/05/Situation-Psychotherapy-in-Austria-2017-10-20.pdf.

[17] Ermann M. (2007). Psychosomatische Medizin und Psychotherapie.

[18] Humer E., Pieh C., Kuska M., Barke A., Doering B.K., Gossmann K., Trnka R., Meier Z., Kascakova N., Tavel P. (2020). Provision of Psychotherapy during the COVID-19 Pandemic among Czech, German and Slovak Psychotherapists. Int. J. Environ. Res. Public Health.

[19] Caparrotta L. (2013). Digital technology is here to stay and the psychoanalytic community should grapple with it. Psychoanal. Psychother..

[20] Johansson R., Ekbladh S., Hebert A., Lindström M., Möller S., Petitt E., Poysti S., Larsson M.H., Rousseau A., Carlbring P. (2012). Psychodynamic Guided Self-Help for Adult Depression through the Internet: A Randomised Controlled Trial. PLoS ONE.

[21] Scharff J.S. (2012). Clinical issues in analyses over the telephone and the internet. Int. J. Psychoanal..

[22] Chherawala N., Shane G. (2020). Up-to-date review of psychotherapy via videoconference: Implications and recommendations for the RANZCP Psychotherapy Written Case during the COVID-19 pandemic. Australas. Psychiatry.

[23] Migone P. (2013). Psychoanalysis on the Internet: A Discussion of its Theoretical Implications for Both Online and Offline Therapeutic Technique. Psychoanal. Psychol..

[24] Bayles M. (2012). Is Physical Proximity Essential to the Psychoanalytic Process? An Exploration Through the Lens of Skype?. Psychoanal. Dial..

[25] Brenes G.A., Ingram C.W., Danhauer S.C. (2012). Benefits and Challenges of Conducting Psychotherapy by Telephone. Prof. Psychol. Res. Prac..

[26] Roesler C. (2017). Tele-analysis: The use of media technology in psychotherapy and its impact on the therapeutic relationship. J. Anal. Psychol..

[27] Huscsava M., Plener P., Kothgassner O.D. (2020). Teletherapy for Adolescent Psychiatric Outpatients: The Soaring Flight of so far Idle Technologies during the COVID-19 Pandemic. Digit. Psychol..

[28] Probst T., Haid B., Schimböck W., Reisinger A., Gasser M., Eichberger-Heckmann H., Stippl P., Jesser A., Humer E., Korecka N. (2021). Therapeutic interventions in in-person and remote psychotherapy: Survey with psychotherapists and patients experiencing in-person and remote psychotherapy during COVID-19. Clin. Psychol. Psychother..

[29] Adler G., Pritchett L.R., Kauth M.R., Nadorff D. (2014). A pilot project to improve access to Telepsychotherapy at rural clinics. Telemed. e-Health.

[30] Elford R., White H., Bowering R., Ghandi A., Maddiggan B., St John K., House M., Harnett J., West R., Battcock A. (2000). A randomized, controlled trial of child psychiatric assessments conducted using videoconferencing. J. Telemed. Telecare.

[31] Fletcher T.L., Hogan J.B., Keegan F., Davis M.L., Wassef M., Day S., Lindsay J.A. (2018). Recent Advances in Delivering Mental Health Treatment via Video to Home. Curr. Psychiatry Rep..

